# Seaweed-Coral Interactions: Variance in Seaweed Allelopathy, Coral Susceptibility, and Potential Effects on Coral Resilience

**DOI:** 10.1371/journal.pone.0085786

**Published:** 2014-01-22

**Authors:** Roberta M. Bonaldo, Mark E. Hay

**Affiliations:** 1 School of Biology, Georgia Tech, Atlanta, Georgia, United States of America; 2 Departamento de Ecologia, IB, Universidade de São Paulo, São Paulo, Brazil; University of New South Wales, Australia

## Abstract

Tropical reefs are in global decline with seaweeds commonly replacing corals. Negative associations between macroalgae and corals are well documented, but the mechanisms involved, the dynamics of the interactions, and variance in effects of different macroalgal-coral pairings are poorly investigated. We assessed the frequency, magnitude, and dynamics of macroalgal-coral competition involving allelopathic and non-allelopathic macroalgae on three, spatially grouped pairs of no-take Marine Protected Areas (MPAs) and non-MPAs in Fiji. In non-MPAs, biomass of herbivorous fishes was 70–80% lower, macroalgal cover 4–9 fold higher, macroalgal-coral contacts 5–15 fold more frequent and 23–67 fold more extensive (measured as % of colony margin contacted by macroalgae), and coral cover 51–68% lower than in MPAs. Coral contacts with allelopathic macroalgae occurred less frequently than expected by chance across all sites, while contact with non-allelopathic macroalgae tended to occur more frequently than expected. Transplants of allelopathic macroalgae (*Chlorodesmis fastigiata* and *Galaxaura filamentosa*) against coral edges inflicted damage to *Acropora aspera* and *Pocillopora damicornis* more rapidly and extensively than to *Porites cylindrica* and *Porites lobata*, which appeared more resistant to these macroalgae. *Montipora digitata* experienced intermediate damage. Extent of damage from macroalgal contact was independent of coral colony size for each of the 10 macroalgal-coral pairings we established. When natural contacts with *Galaxaura filamentosa* were removed in the field, recovery was rapid for *Porites lobata*, but *Pocillopora damicornis* did not recover and damage continued to expand. As macroalgae increase on overfished tropical reefs, allelopathy could produce feedbacks that suppress coral resilience, prevent coral recovery, and promote the stability of algal beds in habitats previously available to corals.

## Introduction

Competition has long been recognized as a critical process shaping the ecology and evolution of species and the structure and function of communities and ecosystems [1], [2], [3]. This is as true for marine as for terrestrial systems, but in marine systems competitive interactions commonly occur among dramatically different species (plants versus animals) rather than primarily among taxonomically similar species. Additionally, anthropogenic impacts are changing the identity of major competitors in marine ecosystems, making periodic re-evaluation of interactions critical. As an example, coral-coral competition was an important topic in the coral reef literature 40 years ago when coral cover was high [4], but more recently, coral cover has dropped, macroalgal cover has increased [5], [6], and studies of coral-macroalgal competition are now critically needed [7], [8], [9], [10], [11].

Unlike in terrestrial and freshwater systems, plant-animal interactions in marine systems include not only consumer-prey interactions but also strong competitive interactions because sessile benthic animals like corals and sponges compete with macroalgae for space and light [8], [10]. On coral reefs, coral-macroalgal competition can be a major determinant of benthic community structure, impacting food web dynamics, topographic complexity of the habitat, biodiversity, and ecosystem function [5], [8], [12]–[15]. Macroalgae compete with corals via multiple direct and indirect mechanisms [8], [13], [16]–[19], but some macroalgae are allelopathic to corals on contact [11], [20], [21].

On healthy reefs, establishment and survival of corals is facilitated by high rates of herbivory that suppress macroalgae and diminish their competition with corals [22]–[24]. In recent decades, however, there have been global-scale declines of herbivorous fishes, corals, topographic complexity, carbonate accumulation, and reef biodiversity in general [5], [6], [25], [26]. The relative roles of global warming, ocean acidification, diseases, loss of herbivores, pollution, and other factors in generating coral loss can be debated [13], [15], [27]–[29]. However, regardless of the causes of reef degradation, disturbed reefs commonly convert from species-rich and topographically complex communities dominated by corals to more species-poor and structurally simplified communities, such as reefs with lower coral cover and more macroalgal cover [6], [25], [30]–[34].

The loss of species, functional groups, and critical biotic processes associated with the shift from coral to macroalgal dominance highlights the need to better understand key mechanisms and processes shaping coral-macroalgal interactions and how these help maintain or degrade the resilience of coral reefs [5], [6]. Macroalgae can have negative physical effects (shading, abrasion) on corals [13], [35], may enhance coral susceptibility to pathogens [17], [18], and may damage corals via allelopathic chemicals [11], [20], [21] or by alterations of the chemical environment to suppress beneficial or enhance detrimental microbes on coral surfaces [7], [10], [19]. Short-term (3-weeks or less) field studies demonstrate that allelopathic metabolites on the surfaces of some macroalgae causes bleaching, and sometimes death, of small coral fragments in field experiments [11], [20], [21], but the consequences for larger, intact coral colonies over longer temporal scales in the field are unknown, as are frequencies of coral contacts with allelopathic versus non-allelopathic macrophytes and how this differs between no-take Marine Protected Areas (MPAs) and unprotected areas (non-MPAs).

In this study, we asked: (1) What is the frequency of coral-macroalgal contacts in the field? (2) Does frequency or extent of coral-seaweed contact differ between replicate MPAs and non-MPAs? (3) Does the probability of contact with common coral species vary as a function of an alga's allelopathic potency? (4) Do common corals differ in the speed and extent of damage caused by allelopathic macroalgae? (5) Does the impact of macroalgal allelopathy vary as a function of coral colony size? (6) Do corals recover from damage caused by allelopathic macroalgae? We address these questions to provide a better understanding of the frequency, dynamics, and outcome of coral-macroalgal contacts on natural reefs that are dominated by corals versus macroalgae. Here we study no-take MPAs and adjacent non-MPAs, but findings may be useful for interactions on degraded versus more intact reef systems regardless of the drivers of coral suppression and macroalgal enhancement.

## Methods

**Figure 1:**
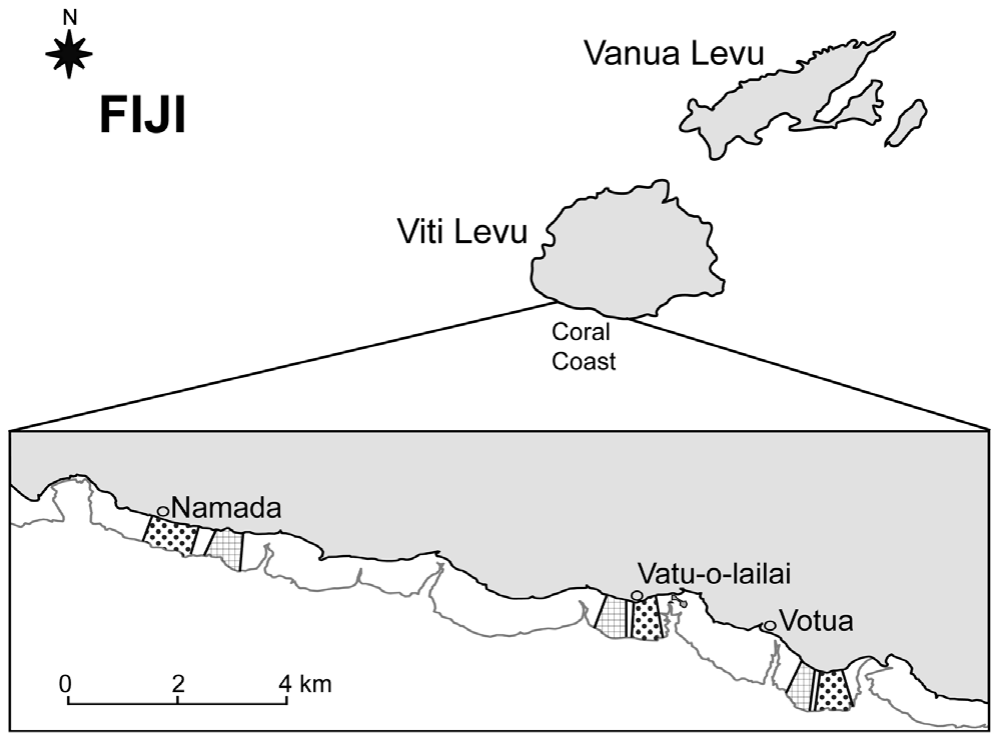
Study sites at Votua, Vatu-o-lailai, and Namada villages along the Coral Coast of Viti Levu, Fiji. Dotted and squared areas correspond to Marine Protected Areas (MPAs) and non-MPAs, respectively, at each site.

Our investigations were undertaken following approval from the Fiji Ministry of Education, National and Heritage, Culture and Arts, Youth and Sports as well as the Korolevu-i-wai district elders. The study was conducted October 2010 to December 2012 on shallow (∼1 m at low tide) fringing reefs with platforms up to 700 m wide along the Coral Coast (18°13.05′S, 177°42.97′E) of Viti Levu, Fiji. We investigated three no-take, Marine Protected Areas (MPAs) and immediately adjacent unprotected areas (non-MPAs) associated with the sites of Votua, Vatu-o-lailai, and Namada Villages (Fig. 1). Paired MPAs and non-MPAs experienced similar physical regimes and the areas we surveyed were separated by about 300 m (at Votua and Vatu-o-lailai) to 600 m (at Namada due to an intervening resort). Sites were separated by 4–11 Km. MPAs were established in 2002 (Vatu-o-lailai, Namada) and 2003 (Votua) and are no-take areas that are well-enforced by local villagers. Shortly after establishment, it is reported that coral cover was low (∼7%), due to a previous bleaching event, and did not differ between MPA and non-MPA areas, while cover of macroalgae was high (35–45%) in both MPA and non-MPA areas [36] (V. Bonito unpublished data).

Coral and macroalgal cover and herbivorous fish biomass were surveyed at high tide in 30 haphazardly placed belt transects 30 m long×4 m wide ( = 120 m^2^ each) running parallel to shore in each MPA and non-MPA ( = 180 transects total). All transects were run between 20 and 300 m seaward of the beach (i.e., in the shoreward half of the reef flat), parallel to the shoreline, and separated by ≥10 m while using shoreline landmarks to avoid resampling the same areas. Given that each MPA and non-MPA area ran for ≥800 m along the beach, transects evaluated less than 2% of any study area, making transects independent and non-overlapping. For fish counts, all roving herbivorous fishes were counted, identified to species, and grouped into 5 cm size classes. Fish lengths were converted to biomass using length-weight relationships [37]. Benthic cover was assessed from photographs taken from 0.5 m above the bottom every 2 m along each transect (i.e., 16 photos per transect). Photos were analysed for % cover of corals and macrophytes using CPC with Excel extension [38]; the program randomly placed 20 points on each photo, and we identified the organism beneath each point (9,000 points/site, 54,000 points over the 6 sites).

Frequencies of coral-macroalgal contacts were assessed with 30 additional 30 m transects per study area, in which each coral colony was visually examined to verify the presence or absence of contact with upright macroalgae. All coral-macroalgal contacts within 2 m of each transect were recorded, interacting corals and macroalgae were identified, frequencies of each coral-macroalgal contact were computed, and each contacted coral was examined for bleaching or death in the area of contact.

The high coral and low macroalgal cover in the MPAs and the inverse pattern in the non-MPAs suggested that frequency of coral-macroalgal contacts would differ dramatically, but we also sampled less intensively to evaluate extent of contact (proportion of colony margin contacted by macroalgae). For this, we focused on a 100×200 m area near the central portions of each site and sampled 10 randomly selected locations within this area. At each location, we measured the perimeter of the closest *Acropora aspera*, *Montipora digitata*, *Pocillopora damicornis*, *Porites cylindrica*, or *Porites lobata* colony as well as the proportion of the perimeter contacted by different macrophytes. We chose these coral species because they were among the most abundant and represent a range of taxonomic groups. If a target coral was not near our sample point, we rotated clockwise scanning a radius of 10 m looking for the first coral of that type. If that coral type did not occur within the 10 m radius, we noted it as missing and moved on to the next station. Some corals (*Acropora, Pocillopora* and *Montipora*) were rare or missing from some non-MPA sites, resulting in total N = 23–49 (pooled across the 5 coral species) in the non-MPAs and 46–50 in the MPAs.

Two experiments evaluated the dynamics of coral-macroalgal interactions in the non-MPA of Votua Village. In the first, *Chlorodesmis fastigiata* and *Galaxaura filamentosa*, which are allelopathic, predictably occur year-round on these reefs, and rapidly bleach several corals following contact [11], [21], were transplanted against five coral species (*Acropora aspera*, *Montipora digitata*, *Pocillopora damicornis*, *Porites cylindrica*, *Porites lobata*) in the field. These corals were abundant along the coast we studied and are within genera that vary in susceptibility to damage from macroalgal contact and allelopathy [11], [21].

One hundred colonies of each coral species with no signs of bleaching or tissue damage were chosen; photographs, including a numeric scale, were taken from the top and side of each coral, and colony surface area was estimated using Image J [39]. Each of the 100 colonies was haphazardly assigned to one of the following five treatments: (n = 20 each): (1) addition of *C. fastigiata*, (2) addition of *G. filamentosa*, (3) addition of a plastic alga to control for shading and contact, (4) addition of a more abrasive control for physical contact (i.e. an artificial alga made of course wool), and (5) a non-manipulation control. Size of coral colonies varied, but variance was similar among treatments. Colony surface area ranged from 55–1,867 cm^2^ for *A. aspera*, 30–3,291 cm^2^ for *M. digitata*, 13–1659 cm^2^ for *P. damicornis*, 44–3,894 cm^2^ for *P. cyllindrica*, and 24–7,591 cm^2^ for *P. lobata*. In total, the experiment consisted of 20 colonies per treatment for each coral species, generating a total of 500 colonies across all coral species.

*Galaxaura filamentosa* was collected by detaching its main stalk at the base and *Chlorodesmis fastigiata* by chiselling off the substratum to which its matted base was attached. Algal size was representative of plants at our field site (∼6 cm tall×4 cm diameter for *C. fastigiata* and ∼20 cm tall×8 cm diameter for *G. filamentosa*). For inert controls, we assembled strips of plastic bags to approximate *C. fastigiata* and lengths of wool yarn to approximate *G. filamentosa*. A rubber band around the holdfast of each natural or artificial alga was attached to a 10 cm wire affixed to a large piece of rubble or other structure adjacent to each experimental coral colony. Treatments were attached upstream of each colony to assure consistent contact. Corals were observed 24 h and 48 h after treatment addition and then on the 2^nd^ and 7^th^ day of every week for 49 days. If damage occurred, corals were photographed, and damaged area assessed at 7–14 day intervals using Image J.

The second experiment assessed coral recovery after removal of allelopathic macroalgae. Thirty colonies of *Pocillopora damicornis* and 30 of *Porites lobata* showing damage due to contact with *Galaxaura filamentosa* were located, and 15 of each species assigned to: (1) macroalgal removal or (2) macroalgal retention (control). Each coral was photographed periodically over 42 days to determine coral condition. Change in area damaged was assessed using Image J. Living corals in contact with *C*hlorodesmis *fastigiata* were rare at this site, preventing an experiment with this species.

### Statistical analyses

Statistical evaluations were conducted in R 3.0.1 [40], except for G tests and calculations of selectivity indices and associated confidence intervals (see below), which were conducted using Excel and resampling add-in version 4.0. Two-way ANOVA (with site and protection status as fixed factors) evaluated differences between MPAs and non-MPAs and among sites for: 1) coral and macroalgal cover, 2) density and frequency of coral-macroalgal contacts (using the number of contacts per transect as the dependent variable), and 3) herbivorous fish biomass (with biomass as dependent variable). Data were log transformed to meet assumptions of normality and homoscedasticity.

The G test for Goodness-of-fit compared the frequency of coral macroalgal interactions in the six study sites with expected frequencies. For the expected frequencies for each coral-macroalgal pair, we first calculated the product of the relative abundance of the given coral species by the relative abundance of the algal species in each study site [41]. This value was then multiplied by the total number of coral macroalgal contacts in each study site to obtain the expected value for the occurrence of that coral-algal contact. Because of the low frequency occurrence of some coral-macroalgal interactions in the field, p-values were obtained by comparing the obtained G-value with that expected from non-parametric randomization tests (10,000 repetitions, [42]). For a better contrast of observed and expected frequencies among all coral-macroalgal pairings, Strauss's Linear electivity index (L, [43]) was calculated for each species pair [44] considering seven abundant coral species and five common macroalgal species known to have strong (*Chlorodesmis fastigiata*, *Dictyota* sp., *Galaxaura filamentosa*) or no allelopathic effects (*Sargassum polycystum* and *Turbinaria conoides*) on corals [11], [21]. Strauss's Linear electivity index was chosen for these analyses because it is straight-forward and commonly used in coral reef studies [45], [46]. Additionally, we ran analyses using Ivlev's electivity, Chesson's alpha, and Scavia's Selectivity indices and found similar patterns, suggesting that our findings were not unduly affected by the choice of a specific index. Data were combined across all study sites, because G-values and difference between the expected and observed frequencies for each coral-macroalgal pairing were similar among sites. Non-parametric bootstrapping procedures generated 95% confidence intervals around the observed L (10,000 randomizations) using the percentile method [42]. 95% CIs that do not overlap zero indicate associations that occur more (positive values) or less (negative values) frequently than expected based on abundances of corals and macroalgae.

To assess if frequencies of interactions of a given macrolagal species with corals in general occurred more, equally, or less than expected at random (i.e. across all coral species), we used the mean L electivity index of each of the seven corals as one independent replicate for that algal species, generated a mean L for corals regarding that macroalga, and tested whether each macroalga was negatively or positively associated with corals in general via a one sample t-test (2-tailed) against an expected value of zero (i.e., no association).

The proportion of the perimeter of coral colonies in contact with macroalgae inside and outside MPAs was compared for each of the three sites with two-way ANOVA (with site and protection status as fixed factors). For each site, two comparisons were done: 1) considering each coral species separately, and 2) pooling all coral species within the same study site. Data were arcsine transformed to meet normality assumptions.

Effects of transplanting algae against corals were evaluated with repeated measures ANOVA, with two between-subject fixed factors (coral species, algal species). Because all control colonies exhibited no damage, controls were excluded from this analysis. Data were square root transformed to meet assumptions of normality and homoscedasticity. To evaluate relationships between coral colony size and size of damage from algal contact, we used Spearman's rank correlation because data did not meet normality assumptions.

We used a range of coral colony sizes in our experiments. To assess whether we might be confounding treatments with colony size effects, we ran a one way ANOVA for each of the five coral species using treatments (*Chlorodesmis fastigiata*, *Galaxaura filamentosa*, Control alga 1, Control alga 2, no alga) as fixed factors and colony size as the variable.

In the experiment involving macroalgal removal, size of damaged area was evaluated with repeated measures ANOVA, with two between-subject fixed factors (coral species, treatment) and one within-subjects factor (damage at day 2, 7, 14, 21, 28 and 42). Data were square root transformed to meet assumptions of normality.

Before parametric tests, data were examined for normality and homogeneity of variances using D'Agostino-Pearson and residual analysis. Where differences were significant in ANOVA, post-hoc tests (Tukey) were used. For the repeated-measure tests, data were also examined for sphericity with the Mauchly's test.

## Results

**Figure 2:**
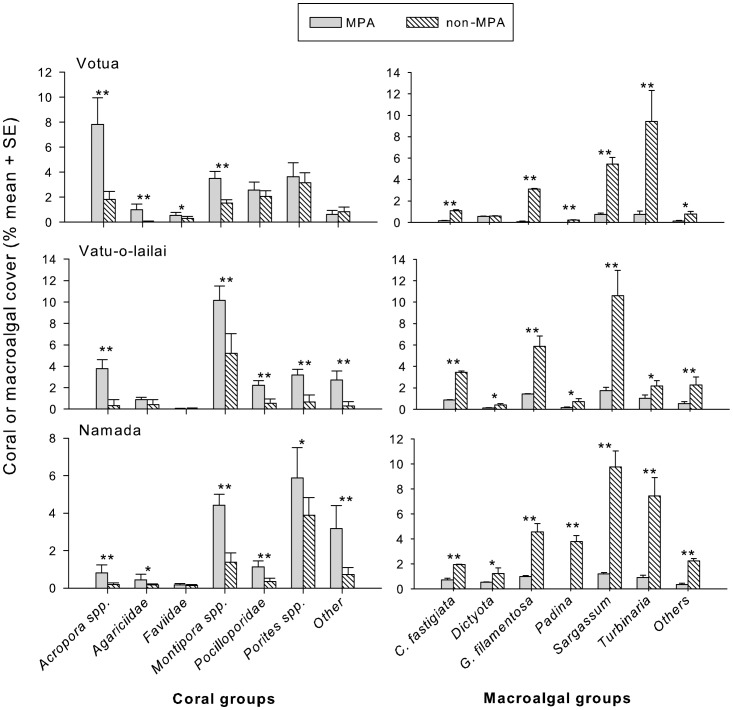
Percent coral and macroalgal cover (mean+SE) in Marine Protected Areas (MPAs) and adjacent non-MPAs associated with three sites along the Coral Coast. * = p<0.05, ** = p<0.01.

The shoreward half of non-MPAs at Votua, Vatu-o-lailai, and Namada had, on average, 4, 9, and 4 times more macroalgal cover and only 32%, 49%, and 42% as much coral cover as their paired MPAs, respectively (Fig. 2). Coral cover was 16% (SE = 4.6), 20% (5.5) and 23% (4.2) in the shoreward areas of the MPAs in Namada, Vatu-o-lali and Votua, respectively, versus 7% (0.8), 10% (1.1) and 7% (4.4) in adjacent areas outside of these MPAs. Similar contrasts for macroalgae were 2% (0.6), 2% (0.9) and 5% (0.6) in the MPAs in Namada, Vatu-o-lailai and Votua versus 28% (3.3), 21% (2.3) and, 27% (3.3) in the non-MPAs, respectively. Of the 7 groups of corals we evaluated, 4–6 were significantly less abundant in non-MPAs versus MPAs at each site. *Acropora* spp. and *Montipora* spp. were significantly more abundant in the MPAs of all sites; Agariciidae, Pocilloporidae, *Porites* spp., and “other” corals were significantly more abundant in two of the three MPAs; Faviid corals were more abundant in one MPA (Fig. 2). No corals were more abundant in the non-MPAs. In contrast to patterns for corals, all 7 algal groups were significantly more abundant in non-MPAs, apart from *Dictyota* in Votua, which was uncommon in both the MPA and non-MPA (Fig. 2).

**Figure 3:**
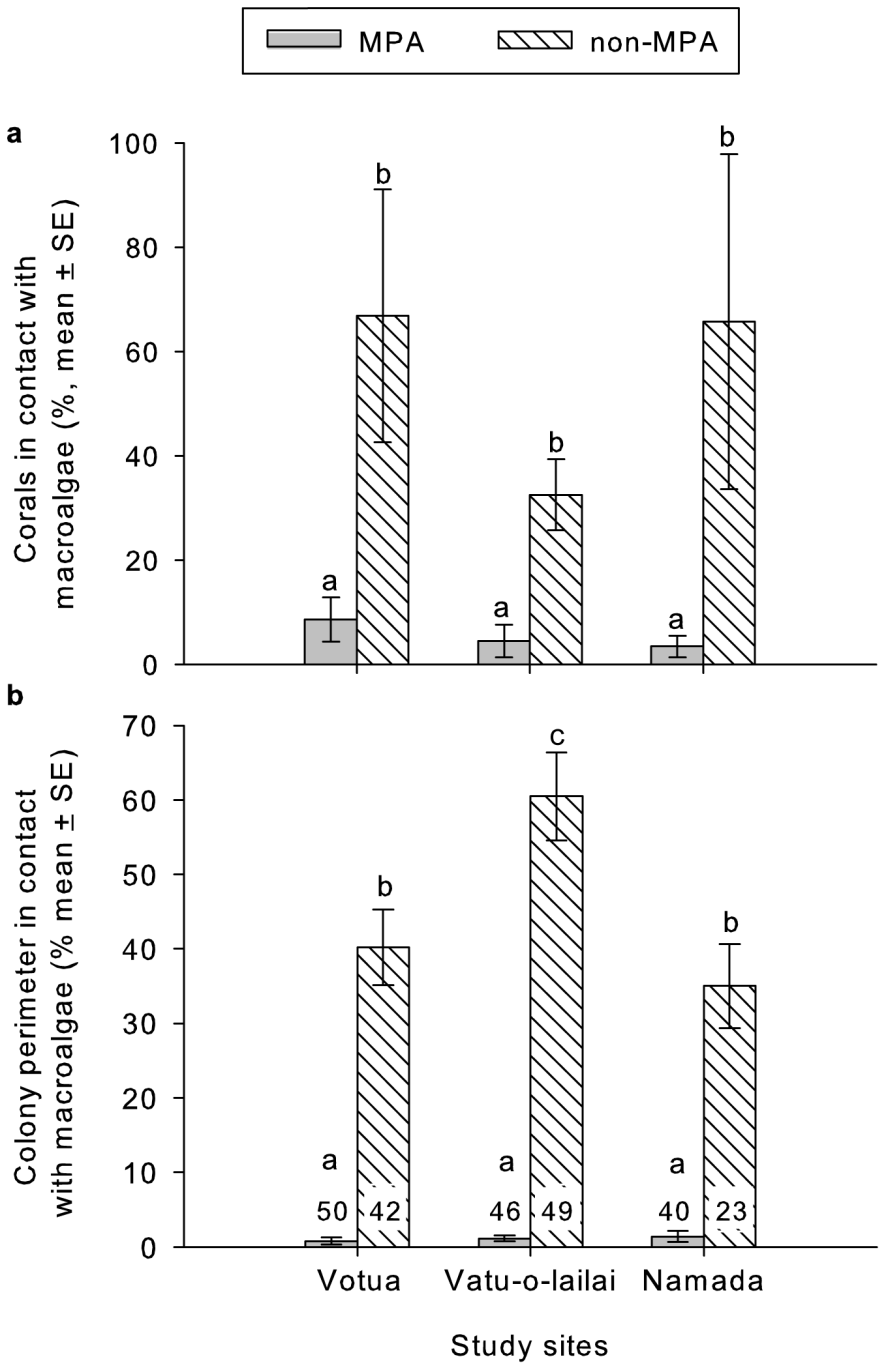
Frequency and extent of macroalgal contact with corals in Marine Protected Areas (MPAs) versus non-MPAs associated with three sites in Fiji. (a) Percentage of individual corals in contact with macroalgae (letters indicate significant groupings) and (b) the proportion of coral colony perimeter (for 5 common coral species pooled) in contact with macroalgae. Numbers provide sample sizes for pooled samples at each location (data by coral type available in Fig. S1). Letters indicate significant groupings (p<0.001 for all contrasts).

**Figure 4:**
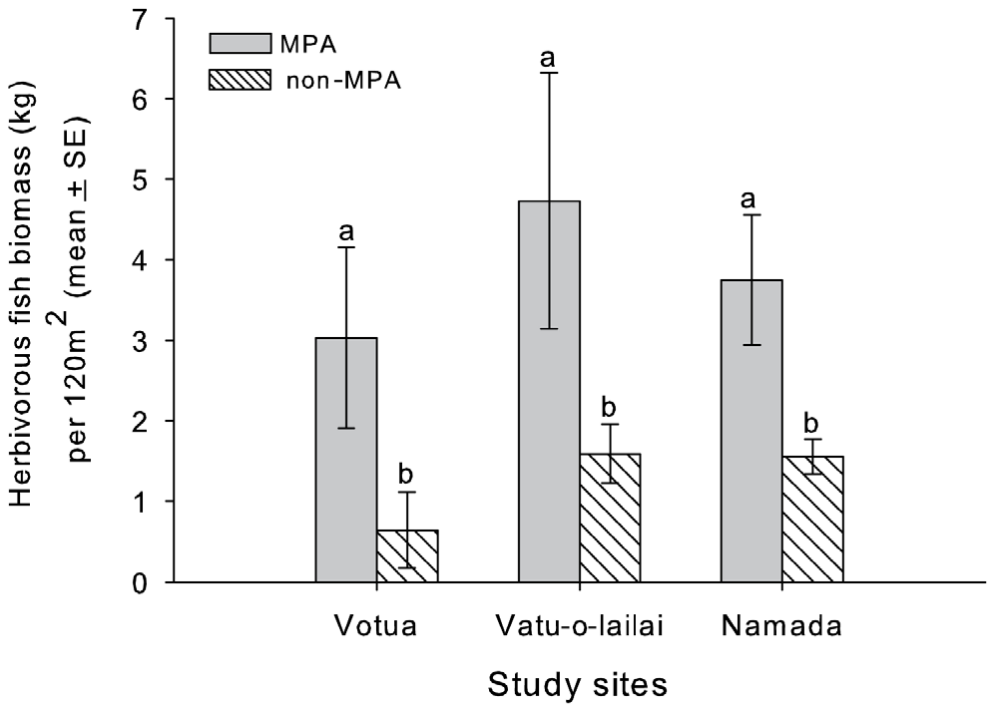
Biomass of herbivorous fishes in MPAs and non-MPAs at three sites along the Coral Coast, Fiji. Letters indicate significant groupings (p<0.05).

Frequency of coral-macroalgal contacts were 5–15 times greater in non-MPAs versus MPAs (p<0.001 for all comparisons, Fig. 3a, Table S1). Similarly, average extent of contact (% perimeter in contacts with macroalgae) in non-MPAs versus MPAs was 23 fold greater at Namada, 41 fold greater at Vatu-o-lailai, and 67 fold greater at Votua (Fig. 3b, S1; p<0.001 for all comparisons). Mean percentage of perimeter in direct contact with macroalgae ranged from 35–63% in the non-MPAs, but only 0.9 to 1.5% in the MPAs. The lesser degree of coral-macroalgal contact in the MPAs may be associated with the mass of herbivorous fishes being 2.5–4 fold higher in MPAs versus non-MPAs (Fig. 4), potentially suppressing macrophyte cover and lessening coral contacts.

Damage to corals found in natural contact with seaweeds in the field varied as a function of algal and coral species (Tables 1, 2). *Sargassum polycystum* and *Turbinaria conoides* never caused visible damage (bleaching or mortality) to any of the 6 coral species they contacted. *Chlorodesmis fastigiata* and *Galaxaura filamentosa* were found in contact with only 3 and 4 coral species respectively; *C. fastigiata* damaged 2 of the 3 corals it contacted and *G. filamentosa* damaged all 4 species it contacted. *Porites cylindrica* was more resistant to macroalgal damage than were the other 5 corals surveyed and was the only coral that exhibited no visual damage to contacts with *C. fastigiata*.

**Table 1:** Frequency of corals with macroalgal contact causing no damage, bleaching, mortality, or mortality with algal overgrowth in three study sites along Coral Coast, Fiji (n≥15 for each coral-macroalga species pair).

Algal species	Coral species	Frequency of coral colonies (%, mean+SE)			
		Undamaged	Bleached	Mortality	Algal overgrowth
*Chlorodesmis fastigiata*	*Montipora digitata*	0	88+22	88+22	88+22
	*Porites cylindrica*	100	0	0	0
	*Porites lobata*	0	100	0	0
*Galaxaura filamentosa*	*Montipora digitata*	0	100	50	0
	*Pocillopora damicornis*	17+5	83+7	0	0
	*Porites cylindrica*	50+4	50+4	0	0
	*Porites lobata*	0	100	23+8	23+8
*Sargassum polycystum*	*Acropora aspera*	100	0	0	0
	*Montipora digitata*	100	0	0	0
	*Pocillopora damicornis*	100	0	0	0
	*Porites cylindrica*	100	0	0	0
	*Porites lobata*	100	0	0	0
	*Seriatopora hystrix*	100	0	0	0
*Turbinaria conoides*	*Acropora aspera*	100	0	0	0
	*Montipora digitata*	100	0	0	0
	*Pocillopora damicornis*	100	0	0	0
	*Porites cylindrica*	100	0	0	0
	*Porites lobata*	100	0	0	0
	*Seriatopora hystrix*	100	0	0	0

**Table 2:** GLM with repeated measures for damage size of coral colonies upon contact with macroalga in two experiments: (1) experimental addition of *Chlorodesmis fastigiata* and *Galaxaura filamentosa* to five coral species, with two between-subject fixed factors (coral species, algal species) and (2) removal of macroalgal contacts with two between-subject fixed factors (coral species, treatment).

Source	*Df*	MS	F	P
**Experiment 1: addition of macroalgal contacts to coral colonies**				
*Between-subject effects*				
Coral	*4*	537.37	4.90	**<0.001**
Alga	*1*	716.80	6.54	0.11
Coral • Alga	*4*	178.44	1.63	0.17
*Within-subject effects*				
Day	*4*	633.27	39.22	**<0.001**
Day • Corals	*16*	87.67	5.43	**<0.001**
Day • Alga	*4*	42.63	2.64	**0.03**
Day • Coral • Alga	*16*	31.05	1.92	0.16
**Experiment 2: removal of macroalgal contacts**				
*Between-subject effects*				
Coral species	*1*	52.64	130.21	**<0.001**
Treatment	*1*	42.79	105.85	**<0.001**
Interaction	*1*	39.10	96.73	**<0.001**
*Within-subject effects*				
Day	*5*	0.97	23.87	**<0.001**
Day • Corals	*5*	1.84	45.11	**<0.001**
Day • Treatment	*5*	1.44	35.22	**<0.001**
Day • Coral • Treatment	*5*	1.32	32.33	**<0.001**

Significant values are highlighted in bold.

**Figure 5:**
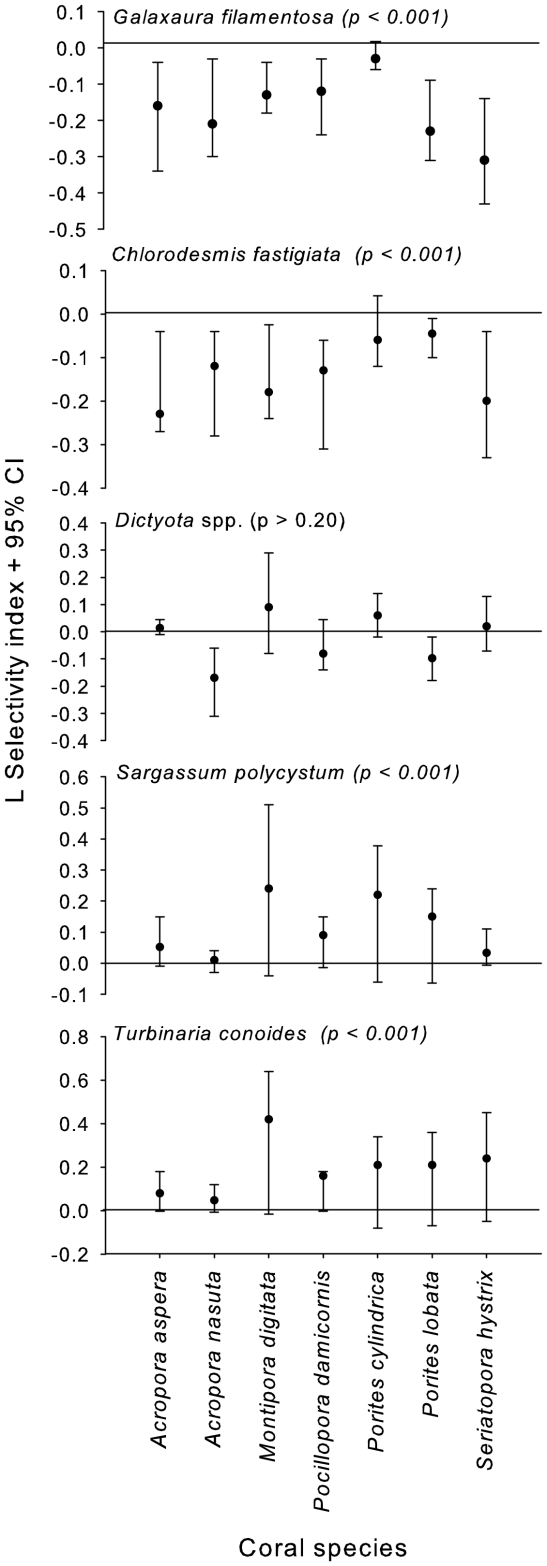
Strauss's Linear Index (L selectivity index) comparing observed versus expected frequency of coral-macroalga contacts for seven abundant corals and five common macroalgae known to have strong (*Chlorodesmis fastigiata*, *Dictyota* sp., *Galaxaura filamentosa*) or no allelopathic effects (*Sargassum polycystum* and *Turbinaria conoides*) on corals [**11]**, [21]. 95% CIs that do not overlap zero indicate associations that occur more (positive values) or less (negative values) frequently than expected based on abundances of corals and macroalgae. P-values by each macroalgal name are from a one-sample t-test evaluating the mean response of all seven corals to that macroalga, using the mean from each coral as one replicate.

Contacts with the allelopathic macroalgae *Chlorodesmis fastigiata* and *Galaxaura filamentosa* were less frequent than expected, given their abundance in the study sites, for 6 of the 7 corals surveyed (Fig. 5). In contrast, coral contacts with the non-allelopathic macroalgae *Sargassum polycystum* and *Turbinaria conoides* were not significantly different than expected for any of the 7 common corals (Fig. 5). Contacts with *Dictyota*, which is allelopathic but seasonal and shows dramatic variance in abundance due to storms or other periodic physical stresses, occurred less frequently than expected for *Acropora nasuta* and *Porites lobata*, but did not differ significantly for the other 5 coral species. Pooling across coral species to evaluate each macrophyte's association with corals in general, coral associations with *C. fastigiata* and *G. filamentosa* were negative (p<0.001), those with *S. polycystum* and *T. conoides* were positive (p<0.001), and associations with *Dictyota* did not differ from random expectations (p>0.20). Table S1 shows how coral-macroalgal frequency of contact varied across sites as a function of coral-macroalga identity. *Turbinaria* and *Sargassum* were commonly contacting corals, especially in the non-MPAs (Table S1).

**Figure 6:**
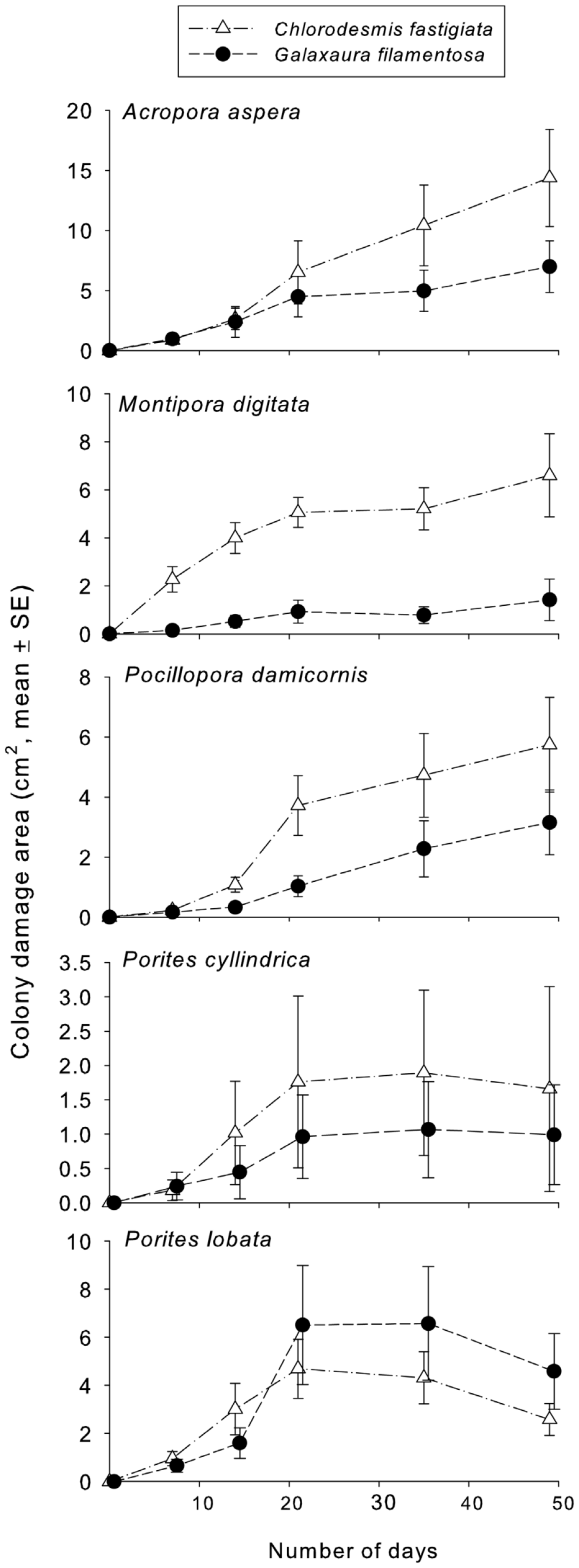
Damaged area (bleached or dead; cm^2^ mean ± SE) over time on corals that had *Chlorodesmis fastigiata* or *Galaxaura filamentosa* transplanted in contact with them. N = 15 for each point. Controls (with inert algal mimics or without algal additions) never experienced bleaching or tissue death and so are not shown.

When *Chlorodesmis fastigiata* and *Galaxaura filamentosa* were transplanted into contact with intact adult colonies of five common corals in the field, both species damaged corals, but damage by *C. fastigiata* tended to be more rapid than damage by *G. filamentosa* for 4 of the 5 corals (Fig. 6). Extent of damage was greatest for *Acropora aspera*, intermediate for *Montipora digitata*, *Pocillopora damicornis*, and *Porites lobata*, and lowest for *Porites cylindrica*. For *M. digitata*, *P. cylindrica*, and *P. lobata*, damage increased for the first three weeks of the experiment, but then stabilized. Damage to *A. aspera* and *P. damicornis* increased throughout the experiment; after day 20, the damage was extending beyond areas of direct algal contact. For these colonies, some areas had no polyps and became overgrown by filamentous algae (localized mortality). In contrast, damage to *M. digitata*, *P. cylindrica*, and *P. lobata* was restricted to bleaching, and occurred only in areas of direct macroalgal contact. None of the control colonies (artificial algae, no algal addition) showed any damage, suggesting chemical effects instead of impacts due to shading or abrasion over this time period.

**Figure 7:**
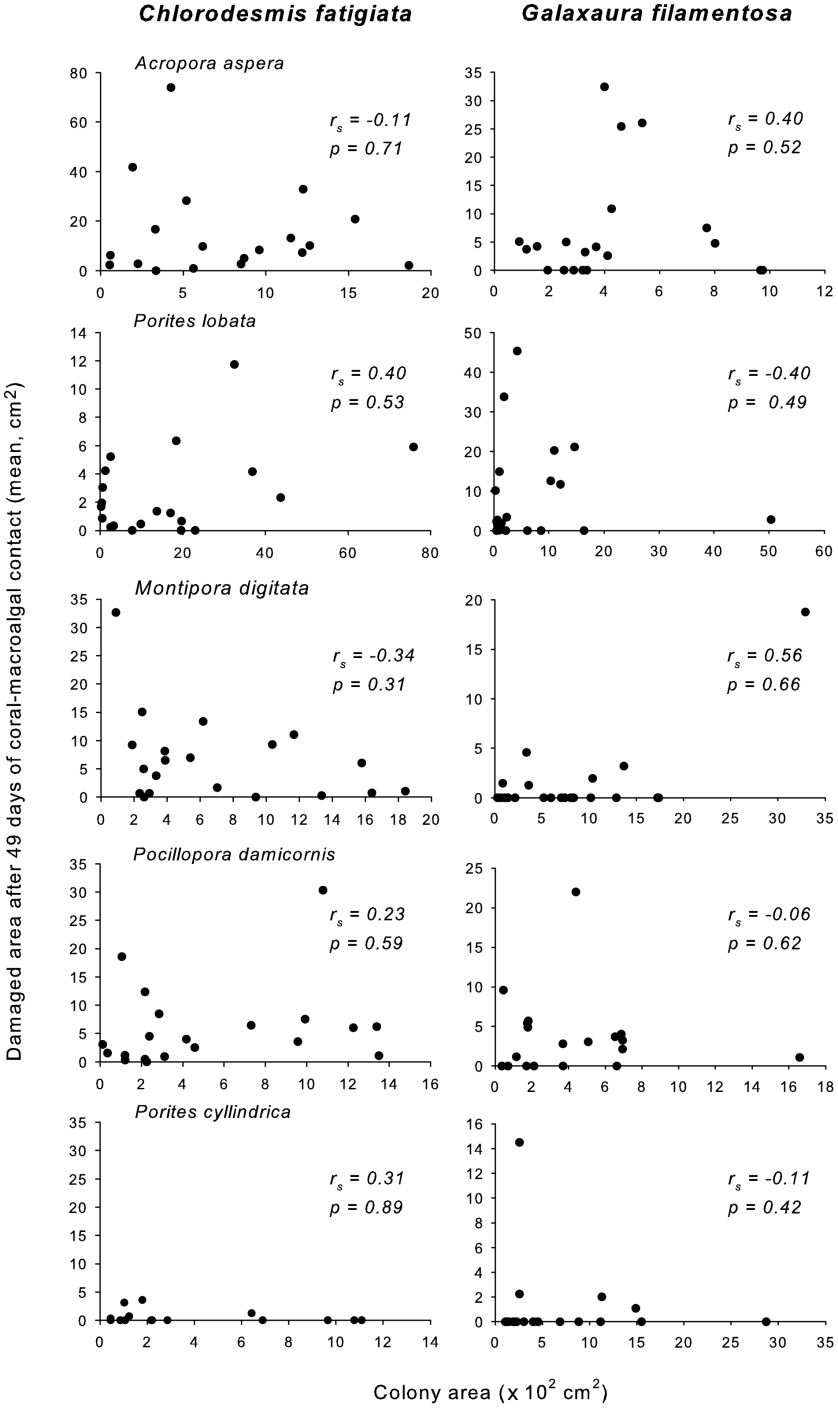
Relationship between colony size, as projected surface area (cm^2^), vs. damage size (cm^2^) of five coral species after 49 days of contact with *Chlorodesmis fastigiata* or *Galaxaura filamentosa*.

Area of coral damage due to contact with *C. fastigiata* or *G. filamentosa* did not vary with coral colony size. Correlations of colony size versus extent of final damage were non-significant (p≥0.31) for each of the 10 coral-algal species pairings (Fig. 7). These contrasts were not confounded by coral colony size; colony size did not differ among any of the five treatments used for each coral-algal pairing (p>0.05 for all species).

**Figure 8:**
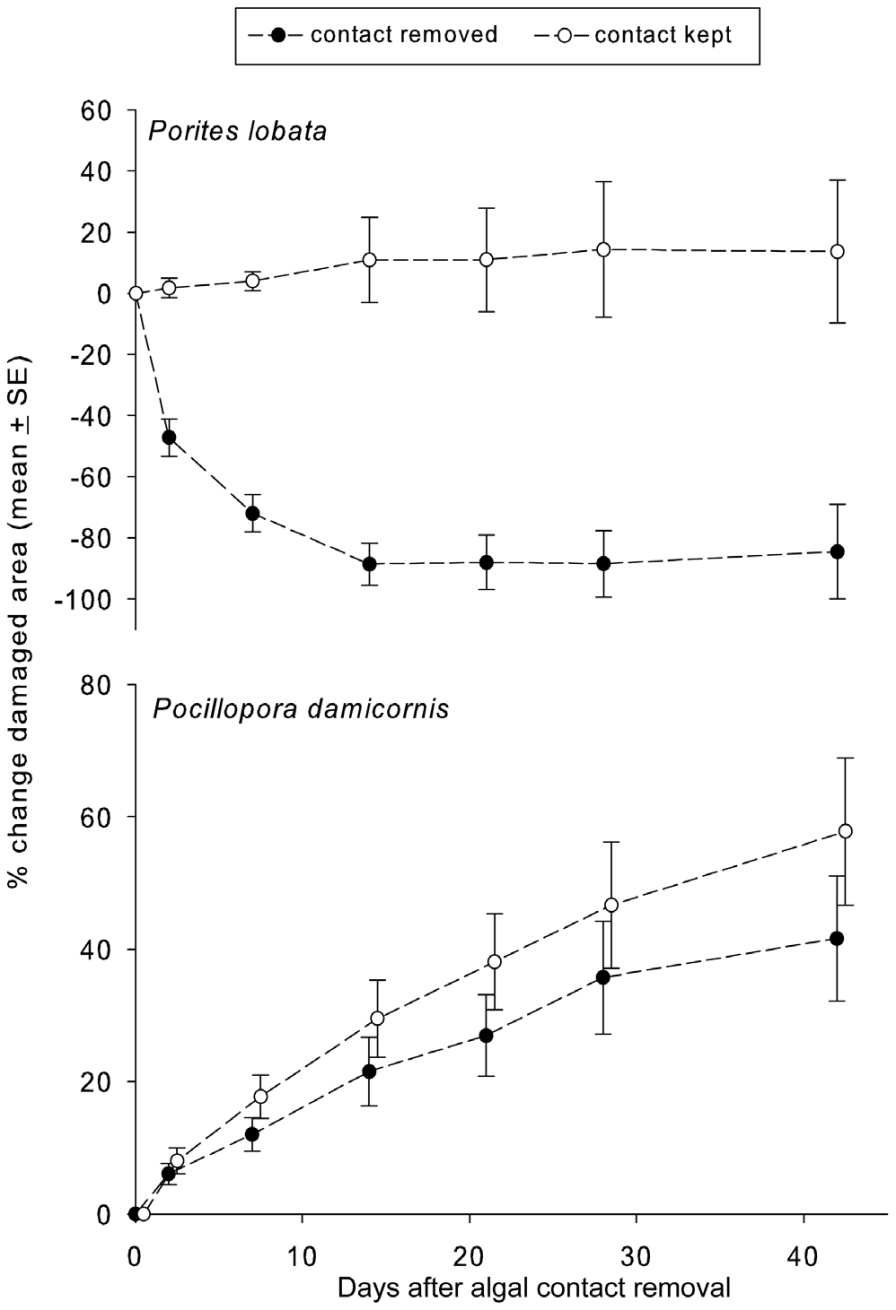
Percent change in size of damaged areas (mean ± SE) over time following removal or retention of natural contacts with *Galaxaura filamentosa*.

Following removal of *Galaura filamentosa* from natural contact with corals in the field, almost all *Porites lobata* colonies recovered within two weeks (Fig. 8), while colonies that retained contact showed no recovery. In contrast, *P. damicornis* experienced no recovery following removal of *G. filamentosa* (Fig. 8, Table 2); damage to colonies both with and without macroalgal contact continued to increase, but those that remained in contact experienced larger areas of damage.

## Discussion

Competition is a key process shaping the structure, diversity, and composition of natural ecosystems [47], [48]. On coral reefs, the replacement of corals by algae is associated with loss of topographic complexity [49], fish abundance and diversity, and reef function [5], [6], [15]. Reefs with abundant macroalgae are less diverse and, thus, more prone to further decline [5], [6]. A negative association between herbivores and macroalgae and between macroalgae and corals is well established [6], [8], [13], [15], but the mechanisms affecting macroalgal-coral interactions and the variance across different coral-macroalgal parings is in need of greater resolution [8], [13], [15]. In our study, coral-macroalgal contacts were 5–15 fold more frequent and 23–67 fold more extensive in non-MPAs versus MPAs. Experimental transplants of macroalgae against common corals demonstrated that allelopathic macroalgae can rapidly damage corals in non-MPAs where they are not removed by herbivorous fishes.

Diverse mixes of herbivorous fishes are critical for suppressing macrophytes, even chemically-rich allelopathic species [50]. Transplants of the common seaweeds from the non-MPAs to MPAs at each of our sites showed the importance of herbivory in suppressing macroalgae in MPAs. When transplanted, the brown macroalgae *Sargassum*, *Turbinaria*, *Padina*, and *Dictyota* were consumed by the unicornfishes *Naso lituratus* and *N. unicornis*; the red alga *Galaxaura* by the parrotfish *Chlorurus sordidus*; and the green alga *Chlorodesmis* by the rabbitfish *Siganus* argenteus. Despite the demonstrated role of herbivores in suppressing macroalgae within MPAs, macroalgae within the non-MPAs could also be facilitated in other ways if bottom-up factors (such as nutrients) operated more strongly there [51]. However, this does not seem to be the case at these reefs. MPA and non-MPA areas at each site are spatially paired, experience similar physical conditions, and when herbivores are experimentally excluded from both MPA and non-MPA sites, macroalgal recruitment and growth is equivalent between sites (demonstrating similar productivity potential); additionally, macroalgae at both sites have equivalent C∶N ratios suggesting equal access to, and use of, nutrients [52]. Finally, data taken shortly after the MPAs were established indicate that coral cover was uniformly low (∼7%) and macrophyte cover uniformly high (35–45%) across all MPA and non-MPAs [36] (V. Bonito unpublished data) prior to the decade of enforced protection in MPAs. These findings collectively support the hypothesis that herbivore removal from the non-MPA sites is a major driver of differences in macrolagal abundance and in frequency and extent of macroalgal-coral contact. This hypothesis also is consistent with the dramatic differences in abundance and diversity of herbivorous fishes between MPAs and non-MPAs at these sites [50].

Macroalgae identified as allelopathic in previous field experiments with small coral fragments [11], [21] were also damaging to large, intact corals in our experiments and the most allelopathic species (*Chlorodesmis fastigiata* and *Galaura filamentosa*) showed significant negative associations with 6 of the 7 common coral species we investigated. In contrast, the non-allelopathic macroalgae (*Sargassum polycystum* and *Turbinaria conoides*) were not negatively associated with any coral; all corals tended toward positive associations with *Sargassum* and *Turbinaria*, and this was significant when pooled across coral species. *Dictyota* is an allelopathic [11], [19] but less persistent macroalga; it was negatively associated with 2 corals (*Acropora nasuta* and *Porites lobata*), but showed no significant association with the other 5 corals. These patterns are consistent with the hypothesis that allelopathic macroalgae may be directly or indirectly suppressing nearby corals, or their recruits, on reefs where macroalgae escape control by herbivores.

For some interactions, damage associated with macroalgal contact extended beyond the areas of direct contact (*Acropora aspera* and *Pocillopora damicornis* contacted by *Chlorodesmis fastigiata*), or damage kept spreading even after the allelopathic alga had been removed (*Galaxaura filamentosa* effect on *P. damacornis*). Such expanding or continuing spread could indicate effects of microbial pathogens or ciliate consumers that attack when seaweeds weaken corals [10], [17]–[19], [53]. Of the 5 coral species we tested against the allelopathic macroalgae *C. fastigiata* and *G. filamentosa*, the branching coral *Acropora nasuta* was most strongly impacted. Acropora *millepora* is also strongly affected by allelopathic macroalgae [11]. If these patterns are typical of other *Acropora* species, many of which play important roles in generating the topographic complexity upon which other reef species depend [54], then suppressing allelopathic macrophytes may be required for retaining reef complexity and function [49], [50].

Macroalgae are known to suppress the settlement and fitness of corals. However, many studies have focused on macroalgae as a group rather than evaluating differences across macroalgal-coral pairings or evaluating how effects might vary with coral size [9], [14], [35], [54]. Our results help fill-in this missing information by demonstrating considerable variance in how different macroalgae affect different corals, that some macroalgae are predictably more damaging than others, that some corals are consistently more susceptible or resistant, that patterns from field experiments are largely consistent with patterns of distribution found in survey data for areas of high versus low macrophyte abundance, and that impacts of allelopathic macroalgae were not ameliorated by coral colony size. One might predict that larger corals, with greater resources to mobilize against competitors, would be less affected, but we found no such pattern.

Even though some macroalgae suppress coral fitness, others, including *Sargassum*, can positively affect corals in some environments by reducing bleaching or protecting recruits from predation [8], [55]. In our study, all coral species exhibited positive electivity values for both *Sargassum polycystum* and *Turbinaria conoides*. Means for individual species were not significantly positive, but evaluations across all coral species demonstrated a positive association for corals in general. On shallow physically stressful reef flats, a canopy of non-allelopathic macroalgae may lessen physical or biological stress and produce benefits that exceed the cost of competition. Positive effects of competitors that suppress stresses also have been documented in other communities [56], [57].

Allelopathic effects of macroalgae on corals have been demonstrated only recently, with a high variance in the potency of different algae and the susceptibility of different corals [11], [20], [21]. Shading, abrasion and direct overgrowth by macroalgae can also damage corals [13], but this was not the case in our study, as biologically inert algal mimics never produced visible damage over the 49 day duration of our experiment. The primacy of allelopathic over physical effects is also suggested by: 1) the lack of negative relationships with large course algae (*Sargassum polycystum* and *Turbinaria conoides*) that should produce greater abrasion and shading, 2) the negative associations and strong and rapid direct effects of *Chlorodesmis fastigiata* and *Galaxaura filamentosa* despite them being smaller and softer than the larger macroalgae, and 3) the identification and strong allelopathic effects of two loliolide derivatives and two acetylated diterpenes from *G. filamentosa* and *C. fastigiata*, respectively, as well as the strong allelopathic effects of surface extracts from these macroalgae [11], [21]. Thus, allelopathy can affect adult corals under field conditions and appears independent of coral size.

Our results, as well as other studies of *Acropora* [11], suggest that *Acropora* spp., the dominant scleractinian group on Indo-Pacific reefs, are among the corals most strongly impacted by contact with allelopathic macroalgae. *Acropora* susceptibility to macrophyte competition is also suggested by its patterns of abundance. *Acropora* was 3–11 times more prevalent in MPAs that supported minimal macroalgae versus non-MPAs that had 4–9 fold higher cover of macroalgae. Although *Acropora* corals can be strongly impacted by physical factors, such as high temperatures and sedimentation [58], the close spatial connection and interspersion of our MPAs and non-MPAs results in similar physical stresses and suggests that differences in *Acropora* cover between these areas may be better explained by differences in reef protection that result in more herbivorous fishes, fewer macrophytes, and reduced coral-macroalgal contacts in the MPAs. *Acropora* species also appear more susceptible than several other common corals to diseases [59], [60], and it is possible that competition with macroalgae exacerbates this susceptibility [17], [18]. It is also possible that seaweeds could act primarily, or additionally, by suppressing recruitment rather than damaging adults; either, or both, of these effects could decrease coral cover over time.

Because coral reef degradation is associated with loss of live coral cover, a number of studies have investigated factors associated with reef resilience and recovery from disturbances [5], [14], [23], [32], [61]. Most studies found that corals could recover, especially if reef fish communities remained intact and suppressed macroalgae [14], [31], [58]. We found that some corals recovered following macroalgal removal while others did not. *Porites lobata* recovered following removal of *Galaxaura filamentosa*, but *Pocillopora damicornis* continued to decline even after *G. filamentosa* was removed, suggesting, as has been noted by others [17], [18], [53], that macroalgae may facilitate coral susceptibility to pathogens, ciliates, or similar enemies. *Acropora* is similarly susceptible to continued spread of tissue damage following contact with some allelopathic macrophytes [11].

Our study and others [14], [15], [50], [62], [63] suggest that well-enforced MPAs can preserve critical ecosystem processes such as herbivory that enhance coral retention or resilience following disturbances. On the reefs we investigated, biomass of herbivorous fishes was 2.5–59 times higher in MPAs than non-MPAs (this study) [50], and mean grazing rates on seven common macroalgae was ∼250–360% faster in MPAs than in non-MPAs [21], [50]. This difference in herbivore mass and activity was associated with dramatically reduced cover of macroalgae, higher cover of corals, and a critical reduction in the frequency and extent of macroalgal-coral contacts.

The present study highlights growing evidence for the potential importance of detrimental interactions between macroalgae and corals to reef dynamics. Greater susceptibility of some coral species to macroalgal contacts may result in a cascading effect throughout the community and could reduce coral resilience in areas dominated by macroalgae. As macroalgae increase on tropical reefs, macroalgal competition and allelopathy could produce feedbacks that suppress coral resilience, prevent coral recovery, and promote the stability of algal beds in habitats previously available to corals.

## Supporting Information

Table S1 **Number of contacts (%) of 7 scleractinian coral species with 5 macroalgal species inside (MPA) and outside (non-MPA) Marine Protected Areas in three study sites in Fiji.** N = number of colonies surveyed in each study location as a function of coral species.
(DOCX)

Figure S1 **Percentage of colony margin that was contacted by macroalgae for each of five common corals in Marine Protected Areas (MPAs) versus non-MPAs associated with three sites in Fiji.** Numbers above bars provide sample sizes (individual coral colonies) for each species at each location. ND = none detected (zero colonies of that species found at that location).
(TIF)
