# Supplementary material for: Seaweed-Coral Interactions: Variance in Seaweed Allelopathy, Coral Susceptibility, and Potential Effects on Coral Resilience
Source: PLoS One. 2014 Jan 22;9(1):e85786. doi: 10.1371/journal.pone.0085786 (PMC3899053; doi:10.1371/journal.pone.0085786)
Supplement: Table S1 — Number of contacts (%) of 7 scleractinian coral species with 5 macroalgal species inside (MPA) and outside (non-MPA) Marine Protected Areas in three study sites in Fiji. N = number of colonies surveyed in each study location as a function of coral species. (DOCX) [file pone.0085786.s001.docx]

| Site | Status | Coral species | % Colonies contacting macroalgae | | | | | | N |
| --- | --- | --- | --- | --- | --- | --- | --- | --- | --- |
|  |  |  | *G. filamentosa* | *C. fastigiata* | *Dictyota spp.* | *S. polycystum* | *T. conoides* | *Others* |  |
| Votua | MPA | *A. aspera* | 0 | 0 | 0 | 5 | 3 | 3 | 65 |
|  |  | *A. nasuta* | 0 | 0 | 0 | 8 | 8 | 0 | 24 |
|  |  | *M. digitata* | 1 | 0 | 1 | 2 | 4 | 3 | 202 |
|  |  | *P. damicornis* | 3 | 0 | 1 | 6 | 12 | 0 | 203 |
|  |  | *P. cyllindrica* | 3 | 11 | 6 | 6 | 10 | 4 | 72 |
|  |  | *P. lobata* | 0 | 0 | 0 | 13 | 24 | 0 | 54 |
|  |  | *S. hystrix* | 0 | 0 | 0 | 7 | 12 | 0 | 69 |
|  | non-MPA | *A. aspera* | 0 | 0 | 0 | 67 | 53 | 80 | 15 |
|  |  | *A. nasuta* |  |  |  |  |  |  | 0 |
|  |  | *M. digitata* | 1 | 2 | 7 | 62 | 48 | 31 | 132 |
|  |  | *P. damicornis* | 3 | 0 | 11 | 41 | 30 | 26 | 108 |
|  |  | *P. cyllindrica* | 1 | 7 | 13 | 30 | 41 | 23 | 90 |
|  |  | *P. lobata* | 16 | 4 | 0 | 58 | 61 | 14 | 57 |
|  |  | *S. hystrix* | 8 | 0 | 0 | 18 | 35 | 15 | 40 |
| Vatu-o-lailai | MPA | *A. aspera* | 0 | 0 | 3 | 1 | 0 | 5 | 76 |
|  |  | *A. nasuta* | 0 | 0 | 0 | 2 | 4 | 2 | 45 |
|  |  | *M. digitata* | 2 | 0 | 3 | 3 | 0 | 2 | 62 |
|  |  | *P. damicornis* | 0 | 0 | 2 | 1 | 2 | 2 | 154 |
|  |  | *P. cyllindrica* | 2 | 3 | 5 | 2 | 6 | 5 | 64 |
|  |  | *P. lobata* | 3 | 3 | 0 | 3 | 8 | 0 | 39 |
|  |  | *S. hystrix* | 1 | 0 | 0 | 2 | 5 | 1 | 83 |
|  | non-MPA | *A. aspera* | 0 | 0 | 0 | 62 | 38 | 31 | 13 |
|  |  | *A. nasuta* | 0 | 0 | 0 | 50 | 63 | 25 | 8 |
|  |  | *M. digitata* | 13 | 7 | 0 | 20 | 33 | 40 | 15 |
|  |  | *P. damicornis* | 3 | 0 | 14 | 42 | 33 | 33 | 36 |
|  |  | *P. cyllindrica* | 0 | 19 | 0 | 22 | 43 | 32 | 37 |
|  |  | *P. lobata* | 11 | 0 | 0 | 49 | 58 | 29 | 45 |
|  |  | *S. hystrix* | 0 | 0 | 0 | 28 | 58 | 8 | 50 |
| Namada | MPA | *A. aspera* | 0 | 0 | 0 | 0 | 0 | 17 | 12 |
|  |  | *A. nasuta* | 0 | 0 | 0 | 0 | 7 | 0 | 15 |
|  |  | *M. digitata* | 0 | 0 | 0 | 5 | 32 | 3 | 37 |
|  |  | *P. damicornis* | 0 | 0 | 2 | 2 | 0 | 9 | 53 |
|  |  | *P. cyllindrica* | 3 | 7 | 0 | 0 | 0 | 7 | 30 |
|  |  | *P. lobata* | 0 | 0 | 0 | 60 | 10 | 30 | 20 |
|  |  | *S. hystrix* | 0 | 0 | 0 | 0 | 3 | 3 | 37 |
|  | non-MPA | *A. aspera* | 0 | 0 | 0 | 0 | 50 | 0 | 2 |
|  |  | *A. nasuta* | 0 | 0 | 0 | 40 | 40 | 20 | 10 |
|  |  | *M. digitata* | 20 | 0 | 0 | 30 | 80 | 50 | 10 |
|  |  | *P. damicornis* | 4 | 0 | 11 | 43 | 32 | 29 | 28 |
|  |  | *P. cyllindrica* | 5 | 14 | 0 | 51 | 41 | 46 | 37 |
|  |  | *P. lobata* | 10 | 5 | 0 | 36 | 49 | 67 | 39 |
|  |  | *S. hystrix* | 3 | 0 | 0 | 25 | 47 | 47 | 36 |

35
