# Supplementary figures and images for: Seaweed-Coral Interactions: Variance in Seaweed Allelopathy, Coral Susceptibility, and Potential Effects on Coral Resilience
Source: PLoS One. 2014 Jan 22;9(1):e85786. doi: 10.1371/journal.pone.0085786 (PMC3899053; doi:10.1371/journal.pone.0085786)

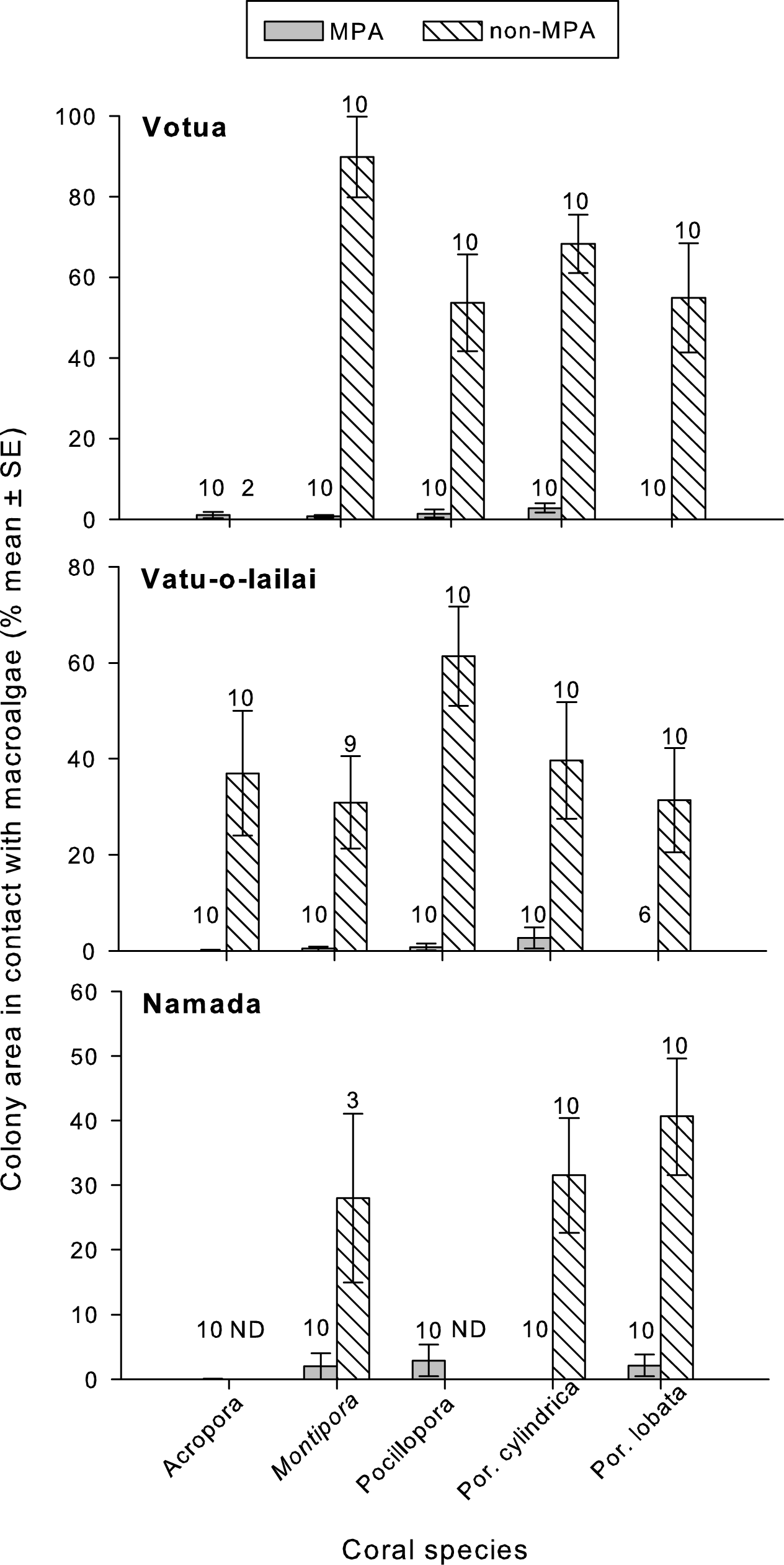

Supplement: Figure S1 — Percentage of colony margin that was contacted by macroalgae for each of five common corals in Marine Protected Areas (MPAs) versus non-MPAs associated with three sites in Fiji. Numbers above bars provide sample sizes (individual coral colonies) for each species at each location. ND = none detected (zero colonies of that species found at that location). (TIF) [file pone.0085786.s002.tif]
